# Draft Genome Sequences of the Enteroinvasive Escherichia coli Strains M4163 and 4608-58

**DOI:** 10.1128/genomeA.01395-14

**Published:** 2015-02-05

**Authors:** Susan R. Leonard, David W. Lacher, Keith A. Lampel

**Affiliations:** Center for Food Safety and Applied Nutrition, Division of Molecular Biology, U.S. Food and Drug Administration, Laurel, Maryland, USA

## Abstract

We report here the draft genome sequences of enteroinvasive Escherichia coli (EIEC) O124:H30 strain M4163 isolated from imported French cheese and EIEC O143:H26 strain 4608-58. The assembled data determined that both strains contain multiple copies of the *ipaH* gene, as well as the pINV A form of the invasion plasmid.

## GENOME ANNOUNCEMENT

As an evolutionary intermediate between pathogenic Escherichia coli and Shigella, enteroinvasive E. coli (EIEC) invades intestinal epithelial cells but has not undergone the extensive gene decay observed in the Shigella genome (1–3). EIEC causes dysentery in infected individuals and has been associated with food-borne outbreaks (4–6). Here, we report the draft genome sequences of two EIEC isolates, M4163 and 4608-58. EIEC M4163 was isolated from imported Camembert cheese associated with a large outbreak in the United States in 1971 (4, 5), and EIEC 4608-58 was obtained from the Walter Reed National Military Medical Center collection (7, 8).

Genomic DNA was extracted from overnight cultures using the DNeasy blood and tissue kit (Qiagen). The sequencing libraries were prepared with the TruSeq DNA sample prep kit (Illumina) and sequenced on the Illumina MiSeq platform, generating 4,488,740 and 1,925,710 paired-end 250-bp reads for isolates M4163 and 4608-58, respectively. The draft genome sequences were assembled *de novo* with CLC Genomics Workbench version 7.0.4 (CLC bio), producing 294 contigs, resulting in a total genome size of 5,093,926 bp and an *N*_50_ of 60,982 bp for isolate M4163, and 266 contigs resulting in a total genome size of 5,043,882 bp and an *N*_50_ of 64,082 bp for isolate 4608-58. The draft genome sequences were annotated using RAST (9) and predicted to contain 5,203 and 4,977 coding sequences for EIEC M4163 and 4608-58, respectively.

Phylogenetic analysis utilizing backbone single-nucleotide polymorphisms (SNPs) from whole-genome sequences revealed that EIEC M4163 clusters with E. coli phylogroup A, while EIEC 4608-58 clusters with phylogroup E (10). Molecular serotyping using the *wzx*, *wzy*, and *fliC* loci indicates that M4163 and 4608-58 belong to serotypes O124:H30 and O143:H26, respectively. The genomes of both strains are approximately 500,000 bp larger than typical Shigella genomes. However, like Shigella genomes, both strains carry multiple copies of the *ipaH* gene. Sequence analysis of the invasion plasmid-associated genes *ipgD*, *mxiC*, and *mxiA* revealed that both EIEC strains possess the pINV A form of the plasmid (11). The 32-kb invasion region on these plasmids shows the greatest sequence similarity to the Shigella boydii type 18 strain CDC 3083-94 (accession no. CP001062) for EIEC M4163 and to the Shigella dysenteriae type 2 strain 1012 (accession no. AAMJ00000000) for EIEC 4608-58. While Shigella isolates are lactose negative, EIEC isolates display variable ability to utilize lactose. EIEC 4608-58 harbors an intact *lac* operon, but the *lac* operon in the EIEC M4163 genome is truncated by an IS*1* element. Consistent with this observation, plating on MacConkey agar demonstrated that EIEC 4608-58 and EIEC M4163 display positive and negative *lac* phenotypes, respectively.

The ability to further characterize these important pathogens will be enhanced with the addition of the sequences generated in this study, particularly since there are very few EIEC genome sequences that are publicly available.

### Nucleotide sequence accession numbers.

The draft genome sequences of E. coli M4163 and 4608-58 were deposited at DDBJ/EMBL/GenBank under the accession numbers JTCN00000000 and JTCO00000000, respectively. The versions described in this paper are JTCN01000000 and JTCO01000000.
